# Understanding barriers to veterinary involvement in dairy calf health management

**DOI:** 10.3389/fvets.2025.1503915

**Published:** 2025-02-12

**Authors:** Kristen Y. Edwards, Angel Abuelo, Stephen J. LeBlanc, Trevor J. DeVries, Michael A. Steele, Joao H. C. Costa, David L. Renaud

**Affiliations:** ^1^Department of Population Medicine, University of Guelph, Guelph, ON, Canada; ^2^Department of Large Animal Clinical Sciences, College of Veterinary Medicine, Michigan State University, East Lansing, MI, United States; ^3^Department of Animal Biosciences, University of Guelph, Guelph, ON, Canada; ^4^Department of Animal and Veterinary Sciences, University of Vermont, Burlington, VT, United States

**Keywords:** analysis, feedback, health records, bovine, veterinarian and farmer

## Abstract

The objectives of this cross-sectional study were to identify barriers to veterinary involvement in calf health and assess knowledge gaps in calf care among American and Canadian bovine veterinarians. A questionnaire was administered to veterinarians, collecting data on demographics, satisfaction with calf health management knowledge, involvement in decision-making, satisfaction with calf health involvement, frequency of calf health record analysis and feedback, topics of interest for further learning, and preferred learning formats. Multivariable logistic regression models were used to assess associations between variables and outcomes. Only 28% of veterinarians frequently reviewed calf health records, and 44% made actionable recommendations after reviewing them. Female veterinarians were more likely than male veterinarians to frequently review calf health records (Odds ratio – OR: 2.9, 95% CI: 1.2–7.3). Additionally, the odds of frequently reviewing records increased with the amount of time spent working with calves (OR: 10.2 per 10% increment, 95% CI: 10.0–10.5). Veterinarians highly satisfied with their knowledge of neonatal calf diarrhea (NCD) prevention were more likely to make recommendations based on records (OR: 11.6, 95% CI: 1.9–72.4). Additionally, those frequently reviewing records were more likely to provide feedback (OR: 15.5, 95% CI: 4.0–60.3). Incomplete records was the most common reason for not reviewing records (60% of respondents) and why actionable recommendations were made less frequently than “most of the time” (67% of respondents). Veterinarians were least confident in their knowledge regarding milk feeding and weaning recommendations but they were interested in learning more about post-weaning nutrition and automated calf feeders. Further, they preferred conference presentations for continuing education. These findings suggest that veterinary involvement in calf health could be improved by facilitating better data capture and enhancing veterinarian knowledge.

## Introduction

Bovine veterinarians serve as key advisors in improving cattle health, welfare, and production (1–3). However, calf health has historically been a secondary focus to the adult herd (4). Almost half of Canadian dairy farmers reported that their veterinarians seldom or never provided feedback based on their calf health records (5). Furthermore, Palczynski et al. (6) reported that many veterinary practices did not include calves as part of their standard herd health visits. Yet, feedback on health data is important to producers. In a survey of cow-calf operators in the United States, 32% of respondents said they would pay for veterinarians to analyze their health records and provide management advice based on the data (7). Further, producers indicated that they wanted practical and tailored actionable recommendations based on their calf health data (6), and when veterinarians provide calf health record analysis and feedback, producers are more likely to record the primary data (5).

The perspectives of veterinarians regarding calf disease management (8), calf welfare (3, 9), and surplus calves (10) have been explored. Veterinarians rank neonatal calf diarrhea as a top priority for the dairy industry (8) and perceive that an important part of their role as a farm advisor is advocating for calf care, including that of surplus calves (10). Further, veterinarians believe that motivating changes in calf care can be achieved through various techniques, including benchmark reporting (10). Veterinarians also desire a more active role in calf health management and welfare (9); however, the barriers to veterinarians’ involvement in calf health are unclear. Ventura et al. (3) reported that veterinarians felt that a lack of knowledge regarding animal behavior and pain compromised their ability to improve dairy cattle welfare. Similarly, a lack of knowledge regarding calf health management may be a barrier to veterinarians’ involvement in calf health on client farms.

The objectives of this cross-sectional study were to investigate barriers to veterinary involvement in calf health and to identify potential knowledge gaps in calf health management among American and Canadian veterinarians. We hypothesized that veterinarians would not be involved in calf health management due to a self-perceived lack of knowledge.

## Materials and methods

A questionnaire was developed (complete questionnaire is available in Supplementary material S1); that contained 31 multiple choice and seven open text questions divided into seven areas of interest: (1) participant and employment demographics; (2) satisfaction with current knowledge of calf health management, specifically regarding neonatal calf diarrhea (NCD) treatment and prevention, bovine respiratory disease (BRD) prevention and treatment, milk feeding and weaning recommendations, colostrum management, and vaccination strategies; (3) involvement in calf health management decision-making for treatment protocol development, feeding and weaning, colostrum management, and vaccination protocols; (4) satisfaction with their current level of involvement with calves on client farms; (5) calf health records analysis and feedback; (6) topics in calf health management that respondents would like to learn more about; and (7) preferred formats of knowledge translation and transfer (KTT). Responses to the questions were collected through an online questionnaire (Qualtrics, Provo, UT) intended to take 5 min to complete. The questionnaire was pre-tested by three veterinarians for clarity. No changes were made following the pre-test.

The questionnaire was electronically distributed to a convenience sample of veterinarians in the United States and Canada from April 2024 until July 2024 through three bovine veterinary associations: American Association of Bovine Practitioners (AABP), Canadian Association of Bovine Veterinarians (CABV), and the Ontario Association of Bovine Practitioners (OABP). No formal sample size calculation was performed; the sample size was based on convenience and willingness of veterinary associations to distribute the questionnaire to their member lists. Eligibility criteria for study participation were residing in the United States or Canada, literate in English or French, being a licensed veterinarian, and having worked with dairy calves, veal calves, or calf ranches within the last 12 months. Individual participant information was anonymous.

Questionnaire data were exported from Qualtrics into Excel (Microsoft Corporation, Redmond, WA) and manually examined for errors and completeness. Multiple choice or open-text responses that contained errors or were incomplete were excluded from the analyses. Variables were renamed and labeled, and multiple-choice responses were converted to numeric values to facilitate analysis. Lastly, responses to open-ended questions about why calf health records were not analyzed or feedback was not provided, and preferred format of KTT, were inductively coded and classified for quantitative analysis. Data were imported into STATA 18.0 SE (StataCorp LP, College Station, TX) for analysis.

Descriptive analyses were performed for all quantitative variables. The frequency of making actionable recommendations after review of calf health records was transformed from an ordinal to a binary outcome (most or all of the time, or less frequently than most of the time). Similarly, due to lack of variability, the frequency of reviewing calf health records was also categorized into a binary outcome: frequent (reviewing records during every or every other herd visit), or infrequent (less than every other herd visit). In total, four multivariable logistic regression models were built to assess the following outcomes: (1) frequency of making actionable recommendations after review of calf health records; (2) frequency of reviewing calf health records; (3) satisfaction of involvement with calves on client farms; and (4) involvement in decision making for milk feeding and weaning protocols. Univariable logistic regression models were built to assess the associations between explanatory variables and these outcomes. The variables screened in univariable analyses for each outcome were identical and included the respondent’s sex, years practicing as a veterinarian, country of residence, percentage of employment hours spent working with calves, type of employment, role in private practice if employed in private practice, number of veterinarians in the private practice, and number of calves serviced by their place of employment. Additionally, the degree of satisfaction with current knowledge in the calf health management areas of NCD prevention, BRD prevention, milk feeding, and vaccination were assessed as explanatory variables. Due to a low frequency of observations for lower degrees of satisfaction, satisfaction was categorized from five levels (extremely dissatisfied, somewhat dissatisfied, neither satisfied nor dissatisfied, somewhat satisfied, extremely satisfied), to three (less than somewhat satisfied, somewhat satisfied, extremely satisfied). In each model, Kendall’s tau-b rank correlation coefficient was calculated for all ordinal explanatory variables to assess pairwise correlations, where variables were considered collinear at tau > |0.6| and *p* < 0.05. Collinear variables included level of knowledge regarding milk feeding and weaning, level of knowledge regarding BRD treatment and BRD prevention, level of knowledge regarding NCD treatment and BRD prevention, level of knowledge regarding NCD treatment and prevention, respondent’s age and number of years practicing as a veterinarian, and the desire to be involved in treatment protocol development and the desire for involvement in vaccination protocol development. As a result, level of knowledge regarding milk feeding, BRD prevention, NCD prevention, years practicing as a veterinarian, and the desire to be involved in treatment protocol development were selected to be offered to the models, as we were more interested in preventative medicine than aspects of treatment.

In each model, continuous variables were assessed graphically for the assumption of linearity and adjusted for non-linearity by assessing if the variable had a quadratic relationship with the outcome. If the relationship was not quadratic or linear, the variable was categorized into quartiles. Any variable with *p* < 0.20 in univariable analysis was included in multivariable models. The multivariable models were built through a backward stepwise elimination process, with variables with *p* ≤ 0.05 retained in the final model, along with variables identified as confounders if their removal led to a > 25% change in the coefficient of a significant variable. The final logistic regression models were assessed using Hosmer-Lemeshow goodness-of-fit tests when continuous variables were included, and Pearson goodness-of-fit tests when only categorical variables were included in the final model.

## Results

The questionnaire was shared with approximately 4,954 veterinarians with some overlap of members in more than one of the associations which we could not quantify, of which 135 responded to the questionnaire, resulting in an approximate response rate of 2.7%. Seven responses were excluded from analysis because the respondent had not worked with calves in the last 12 mo, leading to 128 responses eligible for analysis.

Veterinarian respondents were primarily males (53%), from Canada (62%), 30 to 39 years old (38%), having practiced for 10 to 20 yr., and employed in private practice (80%), of which the majority (61%) were practice owners. The median (range) proportion of working hours spent working with calves in the last 12 mo was 15% (1–99%) and most respondents’ practices serviced between 1,000 to 9,000 calves.

**Figure 1:**
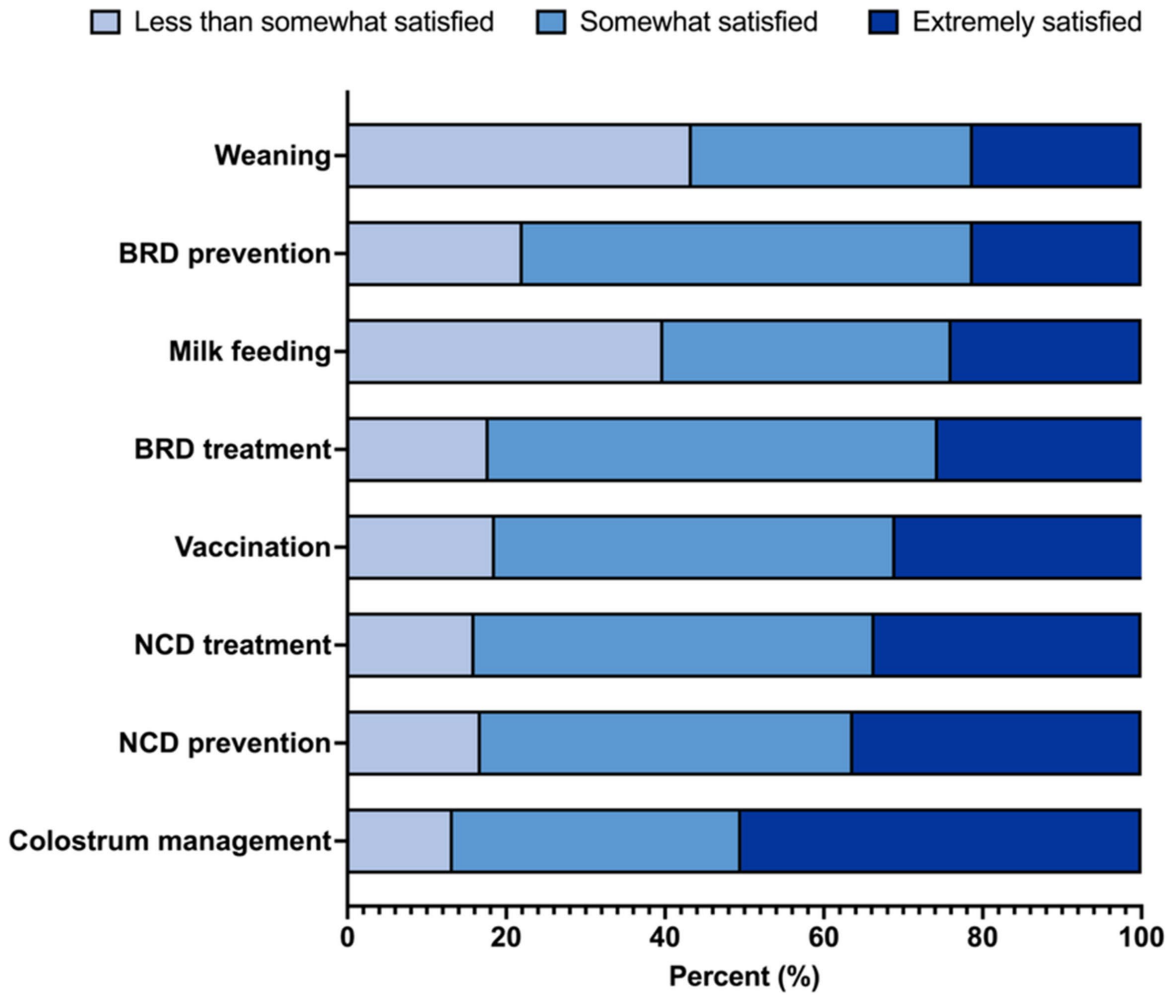
Respondents’ degree of satisfaction with their own knowledge in calf health management areas using data from a questionnaire completed by 128 veterinarians from the United States and Canada. Results are expressed as percentage of responses. NCD, neonatal calf diarrhea; BRD, bovine respiratory disease.

Satisfaction with knowledge across various calf health management areas is presented in Figure 1, with colostrum management, NCD prevention, and NCD treatment being the areas in which veterinarians reported highest satisfaction. Veterinarians were most interested in learning about post-weaning nutrition (74%), followed by automated calf feeders (72%). Additionally, most respondents wanted to receive KTT from oral presentations at conferences (81%), followed by podcasts (56%).

Approximately a quarter (28%) of veterinarians frequently reviewed calf health records and 44% of all respondents reported making actionable recommendations most or all of the time after reviewing calf health records. For those that frequently reviewed calf health records, 81% reported making actionable recommendations most or all of the time. Reasons for why calf health records were not frequently reviewed were provided by 19% (*n* = 15/79) of those who did not frequently review records, with the most common reason being that calf health records were incomplete (60%), followed by a lack of requests from clients to review the records (20%). Twenty percent of respondents who reported making actionable recommendations less than “most of the time” provided reasons, the most common of which was that calf health records were incomplete (67%), followed by a lack of habit for making recommendations based on calf health records (17%).

**Figure 2:**
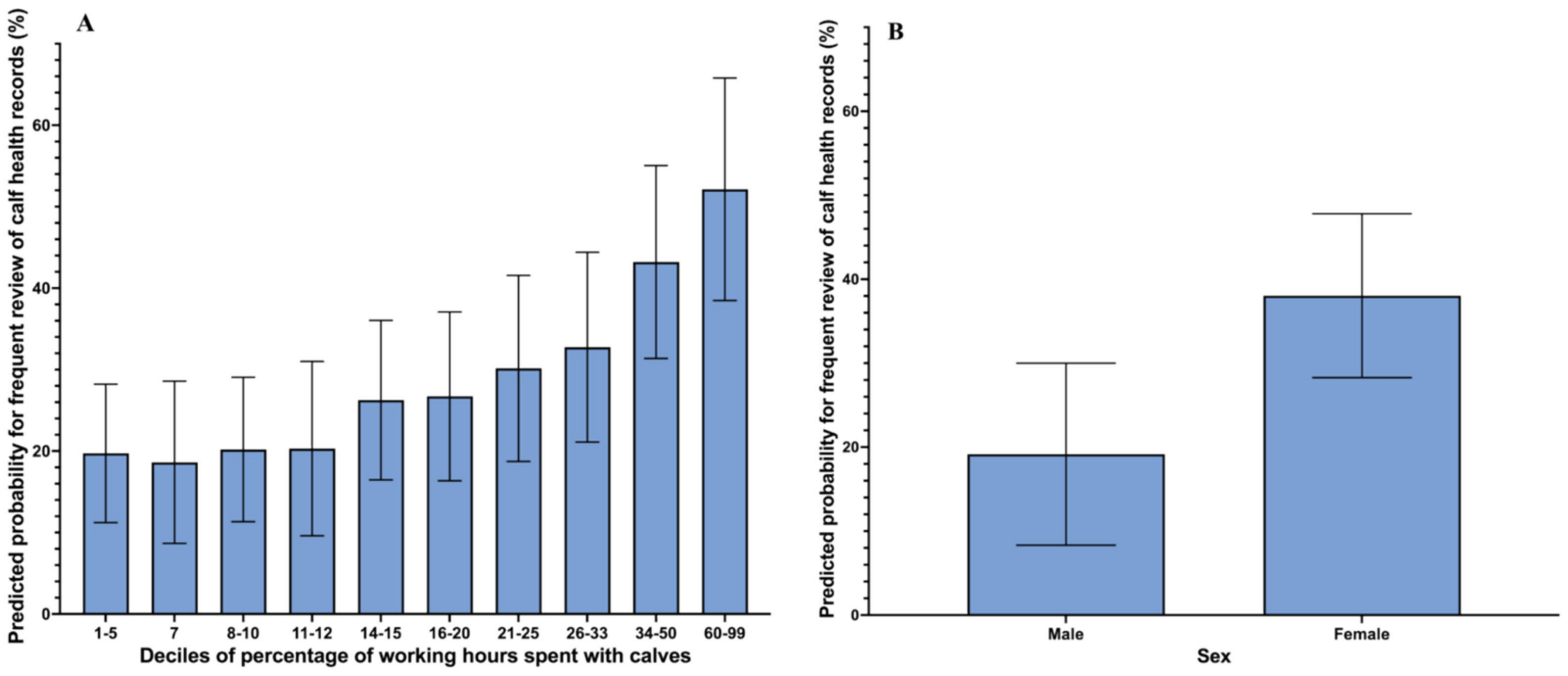
Predicted probability for frequent review of calf health records based on **(A)** the proportion of employment hours spent working with calves, and **(B)** the respondents’ sex. Predicted probabilities are from a logistic regression model using the logit postestimation tool in STATA. The data was obtained from a questionnaire completed by of 128 veterinarians from the United States and Canada. Error bars represent SD. Note that the responses in **(A)** were skewed so the decile categories are not equal percentages of veterinarians’ working time.

Veterinarians had greater odds of frequently reviewing calf health records if they were female (OR = 2.9; 95% CI: 1.2 to 7.3, *p* = 0.02) and if they spent more employment hours working with calves (OR: 10.2 per 10% increment, 95% CI: 10.0–10.5, *p* = 0.02). The probability of frequently reviewing calf health records is shown in Figure 2. Additionally, veterinarians who felt extremely satisfied with their knowledge regarding NCD prevention had 11.6-times greater odds of making actionable recommendations most or all the time compared to those that felt less than somewhat satisfied with their NCD prevention knowledge (Table 1). Also, for every 10% increment of time spent working with calves, the odds of making actionable recommendations most or all the time were 10.5 times greater (Table 1). Lastly, respondents had 15.5-times greater odds of making actionable recommendations most or all the time after reviewing calf health records if they reviewed calf health records frequently compared to those that did not (Table 1).

**Table 1:** Results from a multivariable logistic regression model estimating the odds of making actionable recommendations based on calf health record review using data from a questionnaire completed by 128 veterinarians from the United States and Canada.

Variable	Category	Odds Ratio	Odds Ratio 95% CI	*p* value
Calf health record review	Infrequent^1^ (*n* = 79)	Reference		
	Frequent (*n* = 31)	15.5	4.0–60.3	<0.01
NCD^2^ prevention knowledge	Less than somewhat satisfied (*n* = 19)	Reference		
	Somewhat satisfied (*n* = 53)	4.4	0.8–25.7	0.10
	Extremely satisfied (*n* = 41)	11.6	1.9–72.4	0.01
Percentage of working hours spent with calves	Per 10% increment	10.5	10.2–10.8	<0.01

^1Infrequent review was defined as less than every other herd visit. 2Neonatal calf diarrhea.^

The majority of veterinarians were involved in the decision-making process regarding protocols for vaccination (94%), disease treatment (93%), colostrum management (81%), and feeding and weaning (52%). If given the opportunity, 99% of veterinarians agreed that they wanted to be involved in the decision-making process regarding vaccination, disease treatment (98%), colostrum management (96%), and feeding and weaning (94%). Veterinarians had almost 3 times greater odds of being involved in the decision-making process regarding milk feeding and weaning protocols if they worked in the United States compared to Canada (Table 2) and 6.3 times greater odds if they felt extremely satisfied with their level of knowledge regarding milk feeding recommendations compared to those who were somewhat dissatisfied or extremely dissatisfied (Table 2). Further, veterinarians had 3.2 times greater odds for involvement in milk feeding and weaning protocols if they spent at least 30% of their working hours with calves compared to those that spent 10% or less (Table 2).

**Table 2:** Results from a multivariable logistic regression model estimating the odds of being involved in the decision-making process regarding milk feeding and weaning protocols using data from a questionnaire completed by 128 veterinarians from the United States and Canada.

Variable	Category	Odds Ratio	Odds Ratio 95% CI	*P* value
Country	United States (*n* = 48)	Reference		
	Canada (*n* = 77)	2.7	1.1–10.2	0.03
Milk feeding knowledge	Somewhat or extremely dissatisfied (*n* = 22)	Reference		
	Neither satisfied nor dissatisfied (*n* = 23)	1.8	0.5–6.9	0.40
	Somewhat satisfied (*n* = 41)	1.9	0.6–6.2	0.26
	Extremely satisfied (*n* = 27)	6.3	1.6–23.9	< 0.01
Percentage of working hours spent with calves (quartiles)	1–10% (*n* = 42)	Reference		
	11–15% (*n* = 15)	1.6	0.4–5.7	0.50
	16–29% (*n* = 29)	2.2	0.8–6.5	0.14
	30–99% (*n* = 26)	3.2	1.0–10.2	0.05

Most veterinarians (56%) did not feel satisfied with their level of involvement with calves on client farms. Respondents had lower odds for satisfaction with their level of involvement if they were female compared to male (Table 3) and if they reviewed calf health records every other herd visit, less than every other herd visit, or never, compared to those that reviewed records every herd visit (Table 3).

**Table 3:** Results from a multivariable logistic regression model estimating the odds of respondent satisfaction regarding their level of involvement with calves using data from a questionnaire completed by 128 veterinarians from the United States and Canada.

Variable	Category	Odds Ratio	Odds Ratio 95% CI	*P* value
Sex	Male (*n* = 64)	Reference		
	Female (*n* = 56)	0.3	0.1–0.8	0.01
Frequency of calf health record review	Every herd visit (*n* = 16)	Reference		
	Every other herd visit (*n* = 15)	0.1	0.01–0.5	<0.01
	Less than every other herd visit (*n* = 65)	0.1	0.01–0.4	<0.01
	Never (*n* = 14)	0.04	0.01–0.3	<0.01

## Discussion

This study identified various demographic and knowledge factors associated with veterinarians’ involvement in calf health management. Overall, few veterinarians frequently reviewed calf health records; however, female veterinarians were more likely to review calf health records compared to males. There is little literature evaluating sex-associated differences in health records review among veterinarians, but in human medicine, female physicians spent more time reviewing patient health records than males (11). Further, patients spoke more and disclosed more medical information to female physicians than male physicians (12). In studies evaluating clinical communication among dairy veterinarians and their clients, female veterinarians asked more open-ended questions than male veterinarians (13). These communication differences between sexes may explain why female veterinarians review calf health records more frequently than males, as their communication style may foster a more thorough exchange of information, making them more likely to review calf health records. Additionally, female veterinarians asking more open-ended questions may prompt them to use calf health records as a tool to guide comprehensive care and decision-making. We also detected an association of frequent review of calf health records with greater satisfaction regarding involvement in calf health management. Interestingly, females reviewed calf health records more frequently than males, but also felt less satisfied with their level of involvement with calves on client farms. Sex-based differences in veterinary involvement in youngstock care have not previously been explored. However, females are more often involved and interested in calf care on farm (14, 15) and these sex-associated differences may carry over to the veterinary profession. Specifically, females may desire greater involvement with calves compared to males, leading to a decreased sense of satisfaction with their involvement in calves. It is important to note that these sex-associated differences were independent of age. While females increasingly make up the bovine veterinarian population, especially for practitioners under 40 years old (16), sex was not associated with age or number of years in veterinary practice in our data.

Low satisfaction with the level of knowledge regarding NCD prevention was identified as a barrier to frequently providing feedback on calf health records. Lack of clinical knowledge has been identified as a barrier to providing successful calf health services (17). As NCD is the most common cause of preweaning morbidity and mortality (18), we speculate that veterinarians who feel less confident in their knowledge regarding NCD prevention strategies may not feel sufficiently competent to provide actionable feedback based on calf health records, especially as it relates to the reduction of NCD.

The most common reason for not frequently reviewing or providing feedback on calf health records was that calf health records were incomplete. This is consistent with Doidge et al. (19) who identified that poor quality farm data decreased veterinary confidence in providing feedback on health records. While previous studies have demonstrated that calf health data are often poorly recorded (5, 20), lack of calf health record analysis and feedback from veterinarians influences farmers’ data recording habits and results in incomplete records (5). Moreover, producers believe that veterinarians can motivate improvements in calf heath by making farm-specific data-based recommendations (21) and are more willing to adopt veterinary advice when veterinarians frequently discuss their herd data with them (22). This highlights the importance of veterinarians working with farmers to set up efficient data capture for analysis and feedback to improve both the completeness of calf health data and veterinarians’ involvement in records analysis and feedback.

A greater proportion of working time spent with calves was associated with more frequent calf health record review and feedback, as well as increased involvement in milk feeding and weaning protocol development. Previous studies have identified an association between time and competence, whereby mixed animal veterinarians reported challenges developing competence across multiple species due to insufficient time spent with any given species (23). This may explain why veterinarians who spent more hours working with calves reviewed and reported on calf health records more frequently, as increased competence is associated with increased participation and engagement, as well as decision making (24).

We identified that among calf health management topics, veterinarians felt least satisfied with their knowledge regarding milk feeding and weaning recommendations. Similarly, Palczynski et al. (25) reported uncertainty among both veterinarians and dairy farmers regarding milk feeding and weaning recommendations, thus highlighting the need for nutritional training among veterinarians to competently advise farmers. Additionally, veterinarians were most interested in learning about post-weaning nutrition and automated calf feeders. As milk feeding and weaning knowledge satisfaction was positively correlated with veterinarians’ involvement in milk feeding and weaning decision-making, these findings provide important insight into areas in which bovine veterinarians could be further trained. Lastly, veterinarians identified a preference for learning through oral presentations at conferences as well as podcasts. This is consistent with Gates et al. (26) who also identified conference presentations as the preferred method for veterinary KTT, providing valuable insight for how veterinarians can be most effectively trained in dairy calf nutrition.

There are limitations to consider when interpreting the study results. The first is the low response rate, which might reflect response bias (e.g., to veterinarians with greater interest in calf health), such that the detected associations in this study may differ from the American and Canadian bovine veterinarian populations. As the questionnaire was distributed electronically through three bovine veterinary associations, bovine veterinarians who are not members of those organizations, and those who do not read the membership electronic communications, may not be accurately represented, contributing to a potential nonresponse bias.

The AABP member list accounted for 93% (*n* = 4601/4954) of the questionnaire recipients. As the questionnaire was shared through the AABP newsletter, this may have influenced the low response rate by requiring that veterinarians read the newsletter to become aware of the questionnaire. Lastly, we did not provide incentives, which may also have influenced the response rate. Previous questionnaires administered to bovine veterinarians through AABP have had response rates ranging from 2% (27) to 4% (28). Despite our response rate being within the range of previous studies, the low response rate still serves as a limitation and may be affected by selection bias. Specifically, we had similar respondent demographics to the AABP membership, apart from country of residence. Our respondents were primarily from Canada (*n* = 77/128; 62%) despite only 8% (*n* = 373/4601) of AABP members being Canadian. Due to the anonymous nature of the questionnaire, we were not able to determine the proportion of Canadian respondents that were AABP members versus OABP or CABV members; however, more Canadians may have responded since two of the three bovine organizations were of Canadian origin. As a result, American veterinarians may be underrepresented in this study, thus distorting associations involving country of residence. We also did not explore the veterinary priorities of respondents, which may influence motivations and attitudes toward calf health management and should be investigated in future research. Finally, we did not assess the extent to which client farms relied on other industry advisors for calf care. As a result, veterinarians may not have been the sole advisors, which could also influence their role in calf health management.

This study identified opportunities and challenges to veterinary involvement in dairy calf health management. Less than one third (28%) of veterinarians frequently reviewed calf health records and less than half (44%) of respondents made actionable recommendations most or all of the time after reviewing calf health records. However, there are opportunities to increase veterinary involvement in calf health management by improving veterinarians’ knowledge of NCD prevention, milk feeding, and weaning. Additionally, working with producers to set up efficient calf health data capture for analysis improves calf health record completeness and, subsequently, could be associated with increases in veterinarians’ feedback and involvement. Lastly, we found that veterinarians were most interested in learning about post-weaning nutrition and automated calf feeders and preferred learning through oral presentations at conferences.

## Data Availability

The raw data supporting the conclusions of this article will be made available by the authors, without undue reservation.
