# Supplementary material for: Understanding barriers to veterinary involvement in dairy calf health management
Source: Front Vet Sci. 2025 Feb 12;12:1503915. doi: 10.3389/fvets.2025.1503915 (PMC11861529; doi:10.3389/fvets.2025.1503915)
Supplement: Supplementary file 1 [file Data_Sheet_1.PDF]

Investigating barriers to veterinarian involvement in calf health

---

Start of Block: Consent

Q1 CONSENT TO PARTICIPATE IN RESEARCH
Investigating barriers to veterinarian involvement in calf health

You are invited to participate in a collaborative research survey conducted by Drs. David Renaud and Kristen Edwards from the Department of Population Medicine at the Ontario Veterinary College, University of Guelph, and Dr. Angel Abuelo from Michigan State University. This study is funded by the Natural Sciences and Engineering Research Council of Canada (NSERC) Alliance Grant with the Dairy Farmers of Ontario. Results from the survey will contribute to a research study. If you have any questions or concerns about the research and participation in the survey, or if you would like to receive a summary of results or other information about the study results, please feel free to contact Dr. David Renaud (Study Investigator, Population Medicine, Ontario Veterinary College, University of Guelph) by email: renaudd@uoguelph.ca or telephone: (c) 519-546-8201.

PURPOSE OF THE STUDY
Our goal with this survey is to investigate the barriers to veterinarian involvement in calf health on dairy farms. With information obtained from the survey, researchers will gain a clearer understanding of current veterinarian involvement in calf health, which can be used to identify veterinarian knowledge gaps, develop veterinarian training and education tools, and inform industry best-practice.

PROCEDURES
If you volunteer to participate in this study, we will ask you to do the following: 1. Complete a one-time online survey (~10 minutes using Qualtrics survey platform) regarding your thoughts on working with dairy or surplus calves in veterinary practice. General veterinary practice demographics and personal demographics will also be asked.

POTENTIAL BENEFITS TO PARTICIPANTS AND/OR TO SOCIETY
There are no direct benefits of participation in this study for participants. Participants will be contributing to valuable research that will be publicly presented to the dairy industry and the scientific community. Research findings will help us find ways to improve veterinarian knowledge regarding calf health and well-being. Results will also contribute to identify veterinarian knowledge gaps, development of veterinarian education and training tools, inform industry best-practice recommendation and support consumer confidence in the dairy industry. Findings from the research will be published in academic journals and agriculture publications.

Results will also be presented at scientific and dairy industry conferences and workshops.

CONFIDENTIALITY

Every effort will be made to ensure confidentiality. This survey is anonymous and no personally identifiable information is captured. If identifiers are voluntarily offered in any of the comment fields, they will be removed from the survey and permanently deleted. Additionally, your responses are combined with those of many other respondents and summarized in a report to further protect your anonymity. Only the Principal Investigator of the research will have access to survey data stored on an encrypted USB key in a locked cabinet. Survey data will be kept until publications of survey results are complete, upon which time it will be disposed of. Data with no link to identity will be stored for five years post publications.

POTENTIAL RISKS AND DISCOMFORTS

1. Some questions may make you feel uncomfortable. You can skip any questions you do not wish to answer or quit the entire survey at any time free of repercussions 2. Online data being hacked or intercepted - anytime you share information online there are risks. We are using a secure system to collect survey data and are confident in the system however, we cannot eliminate all risk. Please note that confidentiality cannot be guaranteed while data are in transit over the internet 3. Breach of confidentiality - to minimize the risk that unauthorized individuals inadvertently view survey data we will do the following: 1) Store all electronic data on a password-protected, encrypted computer. 2) Remove and permanently delete any personal identifying information that was voluntarily offered in any of the comment fields of the survey.

PARTICIPATION AND WITHDRAWAL

In order to participate in this research you must be a currently licensed veterinarian that works with livestock. You are under no obligation to participate in this study - you can choose whether to be in this study or not. If you volunteer to be in this study, you may withdraw without consequences of any kind up until the point of submission, but not after, due to the anonymous nature of the data. You may also refuse to answer any questions you don’t want to answer within the survey while remaining in the study with those questions you select to answer. The investigator may withdraw you from this research if circumstances arise that warrant doing so (e.g. responses are unclear). The decision to participate or not will not impact any existing relationships with members of the research team.

RIGHTS OF RESEARCH PARTICIPANTS

You may withdraw your consent at any time and discontinue participation without penalty. You are not waiving any legal rights by agreeing to take part in this study. If you have questions regarding your rights and welfare as a research participant in this study (REB #23-01-021), please contact: Manager, Research Ethics; University of Guelph; reb@uoguelph.ca; (519) 824-4120 (ext. 56606).

INFORMED CONSENT OF RESEARCH PARTICIPANT

I have read the information provided for the study “Investigating barriers to veterinarian involvement in calf health on dairy farms” as described herein. My questions have been

answered to my satisfaction, and I agree to participate in this survey. I am involved in dairy farming, I understand that I can withdraw from the survey up until the point of survey submission and/or not answer specific questions I do not wish to answer, I understand that questions in the survey will cover the following areas: veterinary clinic demographics, personal demographics, and involvement in calf health, and I understand that the identifying information within the survey will be kept confidential.

- ○ Yes, I consent to participate in the research (1)
- ○ No, I do not wish to participate in the research (2)

Page Break

Q2 Are you currently a practicing veterinarian who has done at least some dairy calf, veal calf, or calf ranch work in the past 12 months?

- Yes (1)
- No (2)

Q3 In which country are you located?

- Canada (1)
- USA (2)

Q4 Please select the option that best describes your employment type

- Private practice (1)
- Academia (2)
- Industry (3)
- Government (4)
- Employed directly by a farm (5)
- Other (please specify) (6) ____

Display This Question:

If Please select the option that best describes your employment type = Private practice

Q5 What is your role within private practice?

- ◯ Owner/partner (1)
- ◯ Associate (2)

Display This Question:

If Please select the option that best describes your employment type = Private practice

Q9 Number of veterinarians that provide service to dairy farms, veal farms, or calf ranches at your practice

- ◯ 0 (1)
- ◯ 1 to 4 (2)
- ◯ 5 to 10 (3)
- ◯ > 10 (4)

Q6 Please indicate your sex:

- ◯ Male (1)
- ◯ Female (2)
- ◯ Intersex (3)
- ◯ Prefer not to say (5)

Q7 Please indicate the age category you belong to

- 20 to 29 years (1)
- 30 to 39 years (2)
- 40 to 49 years (3)
- 50 to 59 years (4)
- 60 to 69 years (5)
- > 70 years (6)

Q8 How many years have you been working with livestock as a veterinarian?

- < 1 year (1)
- 1 to 4 years (2)
- 5 to 9 years (3)
- 10 to 20 years (4)
- > 20 years (5)

Q10 Approximate number of calves that your practice services or number of calves at the farm you work at:

- < 1,000 (1)
- 1,000 to 9,000 (2)
- 10 000 to 49,000 (3)
- 50,000 to 100,000 (4)
- > 100,000 (5)

Q11 In the last 12 months, approximately what percentage of your working hours did you spend working with dairy calves, veal calves, or calf ranches?

	0 10 20 30 40 50 60 70 80 90 100
0% ()	

Q12 How satisfied are you with your current knowledge in the following areas:

	Extremely dissatisfied (1)	Somewhat dissatisfied (2)	Neither satisfied nor dissatisfied (3)	Somewhat satisfied (4)	Extremely satisfied (5)
Calf diarrhea prevention (1)	○	○	○	○	○
Calf diarrhea treatment (2)	○	○	○	○	○
Calf respiratory disease prevention (3)	○	○	○	○	○
Calf respiratory disease treatment (4)	○	○	○	○	○
Milk feeding recommendations (5)	○	○	○	○	○
Weaning recommendations (6)	○	○	○	○	○
Colostrum management (7)	○	○	○	○	○
Vaccination strategies (8)	○	○	○	○	○

Q13 Are you involved in the decision-making process on your client's farms regarding the following:

	Yes (1)	No (2)
Calf treatment protocols (1)	○	○
Calf feeding and weaning protocols (2)	○	○
Colostrum management (3)	○	○
Vaccination protocols (4)	○	○

Q14 If given the opportunity, would you like to be involved in the decision-making process on your client's farms regarding the following:

	Yes (1)	No (2)
Calf treatment protocols (1)	○	○
Calf feeding and weaning protocols (2)	○	○
Colostrum management (3)	○	○
Vaccination protocols (4)	○	○

Display This Question:

If If given the opportunity, would you like to be involved in the decision-making process on your cl... = Calf treatment protocols [No]

Q15 Given that you selected no, why do you not wish to be involved in calf treatment protocol development?

____

Display This Question:

If If given the opportunity, would you like to be involved in the decision-making process on your cl... = Calf feeding and weaning protocols [No]

Q17 Given that you selected no, why do you not wish to be involved in calf feeding and weaning protocol development?

____

Display This Question:

If If given the opportunity, would you like to be involved in the decision-making process on your cl... = Colostrum management [No]

Q18 Given that you selected no, why do you not wish to be involved in colostrum management protocol development?

____

Display This Question:

If If given the opportunity, would you like to be involved in the decision-making process on your cl... = Vaccination protocols [No]

Q19 Given that you selected no, why do you not wish to be involved in vaccine protocol development?

____

Q20 Are you satisfied with your level of involvement with calves on your client's farms?

- ○ Yes (4)
- ○ No (5)

Display This Question:

If Are you satisfied with your level of involvement with calves on your client's farms? = No

Q27 Given that you said no, what are the reasons that your involvement with calves is less than you would like?

____

Q21 With respect to farms you work with, with what frequency do you review their calf health records?

- ○ Every farm visit (1)
- ○ Every other farm visit (2)
- ○ Never (3)
- ○ Other (please specify) (4) ____

Display This Question:

If With respect to farms you work with, with what frequency do you review their calf health records? = Never

Q22 Given that you selected never, why do you not review calf health records?

____

Q23 With respect to farms you work with, with what frequency do you make actionable recommendations based on their calf health records?

- Always (1)
- Most of the time (2)
- About half the time (3)
- Seldom (4)
- Never (5)

Display This Question:

If With respect to farms you work with, with what frequency do you make actionable recommendations b... = Seldom

And With respect to farms you work with, with what frequency do you make actionable recommendations b... = Never

Q24 Given that you selected seldom or never, why do you not make actionable recommendations based on calf health records?

____

Q25 What topics within calf management would you like to learn more about? Please select all that apply:

- ☐ Pre-weaning nutrition (1)
- ☐ Post-weaning nutrition (2)
- ☐ Weaning (3)
- ☐ Disease prevention (4)
- ☐ Disease treatment (5)
- ☐ Housing and environment (6)
- ☐ Automated calf feeders (7)
- ☐ Other (please specify) (8) ____

Q26 What format would you like to receive calf-related educational materials? Please select all that apply.

- ☐ Conference presentations (1)
- ☐ Hands-on wet labs (2)
- ☐ Electronic newsletters (3)
- ☐ Podcasts (4)
- ☐ Other (please specify) (5) ____

End of Block: Consent
